# Genome-Wide Analysis of *NBS-LRR* Genes From an Early-Diverging Angiosperm *Euryale ferox*

**DOI:** 10.3389/fgene.2022.880071

**Published:** 2022-05-13

**Authors:** Lan-Hua Qian, Jia-Yi Wu, Yue Wang, Xin Zou, Guang-Can Zhou, Xiao-Qin Sun

**Affiliations:** ^1^ Suzhou Polytechnic Institute of Agriculture, Suzhou, China; ^2^ Institute of Botany, Jiangsu Province and Chinese Academy of Sciences, Nanjing, China; ^3^ Seed Administrative Station of Suzhou, Suzhou, China; ^4^ College of Agricultural and Biological Engineering (College of Tree Peony), Heze University, Heze, China

**Keywords:** *Euryale ferox*, *NBS-LRR* genes, *R* genes, phylogenetic analysis, evolution

## Abstract

*NBS-LRR* genes are the largest gene family in plants conferring resistance to pathogens. At present, studies on the evolution of *NBS-LRR* genes in angiosperms mainly focused on monocots and eudicots, while studies on *NBS-LRR* genes in the basal angiosperms are limited. *Euryale ferox* represents an early-diverging angiosperm order, Nymphaeales, and confronts various pathogens during its lifetime, which can cause serious economic losses in terms of yield and quality. In this study, we performed a genome-wide identification and analysis of *NBS-LRR* genes in *E. ferox*. All 131 identified *NBS-LRR* genes could be divided into three subclasses according to different domain combinations, including 18 *RNLs*, 40 *CNLs*, and 73 *TNLs*. The *E. ferox NBS-LRR* genes are unevenly distributed on 29 chromosomes; 87 genes are clustered at 18 multigene loci, and 44 genes are singletons. Gene duplication analysis revealed that segmental duplications acted as a major mechanism for *NBS-LRR* gene expansions but not for *RNL* genes, because 18 *RNL* genes were scattered over 11 chromosomes without synteny loci, indicating that the expansion of *RNL* genes could have been caused by ectopic duplications. Ancestral gene reconciliation based on phylogenetic analysis revealed that there were at least 122 ancestral *NBS-LRR* lineages in the common ancestor of the three Nymphaeaceae species, suggesting that *NBS-LRR* genes expanded slightly during speciation in *E. ferox*. Transcriptome analysis showed that the majority of *NBS-LRR* genes were at a low level of expression without pathogen stimulation. Overall, this study characterized the profile of *NBS-LRR* genes in *E. ferox* and should serve as a valuable resource for disease resistance breeding in *E. ferox*.

## Introduction

*Euryale ferox* (prickly waterlily) is an annual aquatic plant in tropical and subtropical regions of Southeast and East Asia and is the only species in the Euryale genus in the Nymphaeaceae family. It is cultivated as a nutritious food due to its high starch content; additionally it is also a common Chinese traditional medicine which can treat depression and diabetes mellitus (Ahmed et al., 2015; Wu et al., 2017; Huang et al., 2018; Liu et al., 2018). However, the productivity of *E. ferox* is threatened by various pests and microbial pathogens, including fungi, bacteria, and viruses. The diseases caused by these pathogens pose serious threats to not only plant growth and productivity but also the quality of the edible tissues. However, up to now, no functional disease resistance genes (*R* genes) have been cloned from *E. ferox*.

In order to cope with pathogen invasion, plants have developed sophisticated immune systems during the long-term evolutionary process. *R* genes in plants, which play a core role of the immune system, detect pathogens and trigger downstream resistance response, frequently accompanied by hypersensitive reactions (HR) (Cui et al., 2015; Jones et al., 2016; Wang and Chai, 2020). The nucleotide-binding site-leucine-rich repeat (*NBS-LRR*) genes are the largest *R* gene family in plants. To date, more than 60% of the functional resistance genes cloned from angiosperms belong to the *NBS-LRR* gene family, which can provide resistance to bacteria, fungi, viruses, nematodes, and other pathogens (Kourelis and van der Hoorn, 2018). *NBS-LRR* genes originated in the common ancestor of all green plants, and diverged early into different subclasses (subfamilies) with different domain combinations along plant evolution (Xue et al., 2012; Shao et al., 2019). According to the N-terminal domain, angiosperm *NBS-LRR* genes can be divided into three subclasses: *TIR*-*NBS-LRR* (*TNL*), *CC*-*NBS-LRR* (*CNL*), and *RPW8*-*NBS-LRR* (*RNL*) (Shao et al., 2016). The majority of *TNL* and *CNL* proteins usually serve as pathogen detectors (Kourelis and van der Hoorn, 2018). *NBS-LRR* proteins are usually in a signaling-competent yet autoinhibited state, with LRR domain folding back onto the central NACHT domain (Hu et al., 2013). After recognizing pathogen effectors injected into the host cell, *NBS-LRR* proteins are activated, and NBS domain underwent conformational alterations, with exposed N-terminal domains to trigger downstream hypersensitive reactions, finally eliciting apoptosis of infected cells to suppress the pathogens transmission and proliferation (Andersen et al., 2018). The low expression of *NBS-LRR* genes are considered a normal stage and logically makes sense, otherwise activated HR would cause apoptosis all over plant organs and tissues. The *RNL* subclass can be divided into two small and ancient subclasses, namely *ADR1* and *NRG1*, which can transduce immune signals and function downstream of ‘sensor *NBS-LRR*’ (sNLR) activation, so it was called ‘helper *NBS-LRR*’ (hNLR) (Collier et al., 2011; Shao et al., 2016; Zhong and Cheng, 2016). Furthermore, *NRG1* proteins have advantages in *TNL*-mediated immune signaling (Peart et al., 2005; Qi et al., 2018; Castel et al., 2019; Wu et al., 2019). Recent studies indicated that as Ca2+-permeable channels, *CNL* and *RNL* proteins provoked immune response and cell death (Bi et al., 2021; Jacob et al., 2021).

Since the genome-wide study of *NBS-LRR* in *Arabidopsis thaliana* and rice early in the 21st century, *NBS-LRR* genes have been identified and analyzed in dozens of plant genomes (Meyers et al., 2003). These studies have found that the number of *NBS-LRR* genes varies greatly among different species, ranging from dozens to hundreds (Liu et al., 2021; Luo et al., 2012; Qian et al., 2017; Shao et al., 2014; Xue et al., 2020; Zhang et al., 2016; Zhou et al., 2020). These results provided abundant data resources for studying the structure, mechanism, and evolution of *NBS-LRR* genes. However, the current understanding of *NBS-LRR* gene evolution in angiosperms is mainly derived from studies of monocots and dicots, and the research on *NBS-LRR* genes in the basal angiosperms are limited to *Amborella trichopoda* (Shao et al., 2016). Recently, the genomic data of *E. ferox* provided not only important data resources for the study of disease resistance genes in this species but also a new research object for the in-depth study of the characteristics and evolution pattern of *NBS-LRR* genes in basal angiosperm species (Yang et al., 2020). In this study, *NBS-LRR* genes in the *E. ferox* genome were comprehensively identified and analyzed, which laid a foundation for the subsequent screening of disease resistance resources and the cloning of disease resistance genes and provided a theoretical basis for the evolution of *NBS-LRR* genes in angiosperms.

## Materials and Methods

### Data Source

The genome sequence and annotation files of *E. ferox* were downloaded from the Comparative Genome (CoGe) database (https://genomevolution.org/CoGe/GenomeInfo.pl?gid=56574). The RNA-seq data of *E. ferox* was downloaded from the National Center for Biotechnology Information (NCBI) database (https://www.ncbi.nlm.nih.gov/sra/?term=SRR9650346). The *NBS-LRR* genes in *Nymphaeaceae colorata* and *N. thermarum* were downloaded from the ANNA database (http://compbio.nju.edu.cn/app/ANNA/) (Liu et al., 2021).

### Identification of *NBS-LRR* Genes

*NBS-LRR* genes were identified in the *E. ferox* genome as described previously (Shao et al., 2015). Briefly, an HMM search was first conducted for the protein sequences of *E. ferox* with the amino acid sequence of the NB-ARC domain (Pfam: PF00931) as a query, with a threshold expectation value of 1.0. Meanwhile, BLASTp search was performed towards the protein sequences of *E. ferox* using the sequence of the HMM profile of the NB-ARC domain (E-value = 1.0). Then, all hits obtained using the two methods were merged, and the redundant hits were removed. In order to confirm that blast hits contained the NBS domain, a round of HMMscan was conducted for all the obtained proteins using a more strict threshold expectation value (E-value set to 0.0001). All non-redundant candidate sequences were submitted to an online tool—the Conserved Domains Database at NCBI (CDD; http://www.ncbi.nlm.nih.gov/Structure/cdd/wrpsb.cgi) to further verify the CC, TIR, RPW8, LRR, and other integrated domains.

### Chromosomal Anchoring of *NBS-LRR* Genes in *Euryale ferox* Genome

To anchor each of the *NBS-LRR* genes to specific positions on chromosomes of the *E. ferox* genome, the annotation file recording the positions of all genes was used, and the genomic locations of all the *NBS-LRR* genes were extracted from the file. The flanking regions of each *NBS-LRR* gene (a 250 kb window upstream and downstream, respectively) were searched for the presence/absence of other *NBS-LRR* genes. If another *NBS-LRR* gene was detected within the flanking regions, the two *NBS-LRR* genes were considered as located in the same *NBS-LRR* gene cluster (Ameline-Torregrosa et al., 2008), otherwise the gene initially analyzed should be considered as a singleton.

### Sequence Alignment and Phylogenetic Analysis

The sequence alignment of the NBS domain and phylogenetic analysis were conducted as described by Zhang et al. (2016). First of all, amino acid sequences of the NBS domain from all the identified *NBS-LRR* genes were extracted and aligned by the ClustalW software integrated into MEGA 7.0 (Kumar et al., 2016), with default settings and then manually corrected to achieve a better alignment. Sequences that were too short or extremely divergent were removed to prevent interference with the alignments and subsequent phylogenetic analysis. Phylogenetic analysis was carried out with IQ-TREE using the maximum likelihood method after selecting the best-fit model using ModelFinder (Nguyen et al., 2015; Kalyaanamoorthy et al., 2017). Support values of branches were calculated by UFBoot2 (Minh et al., 2013).

### Analyses of Gene Synteny and Gene Duplication Types

Paralogues in the *E. ferox* genomes were first examined using pair-wise all-against-all BLAST (Altschul et al., 1990), then the obtained results and the GFF annotation file were subjected to MCScanX to evaluate the microsynteny relationships between paralogues. Syntenic genes were considered as the results of whole genome duplications or chromosomal segmental duplications (Wang et al., 2012). Microsynteny relationships were displayed using TBtools (Chen et al., 2020).

## Results

### Genome-wide Identification of *NBS-LRR* Genes in *Euryale ferox*

Altogether 131 *NBS-LRR* genes (40 *CNL*s, 73 *TNL*s, and 18 *RNL*s) (Supplementary Table S1) were identified from the *E. ferox* genome, accounting for approximately 0.6% of all annotated genes. The number of *NBS-LRR* genes in the *E. ferox* genome was smaller than those in Brassicaceae, Solanaceae, Leguminosae or Poaceae as reported by previous studies (Luo et al., 2012; Shao et al., 2014; Zhang et al., 2016; Qian et al., 2017), which may have been due to a convergent contraction in *NBS-LRR* genes during adaptation to an aquatic lifestyle, and was consistent with the report by Liu et al. (Liu et al., 2021). Notably, of three subclasses, the number of *RNL* genes is higher than that in most investigated angiosperms, and *TNL*s account for a larger proportion, suggesting that *E. ferox* tends to employ *TNL*-mediated defense when confronting pathogens.

### Diversity of *NBS-LRR* Gene Structure

**FIGURE 1:**
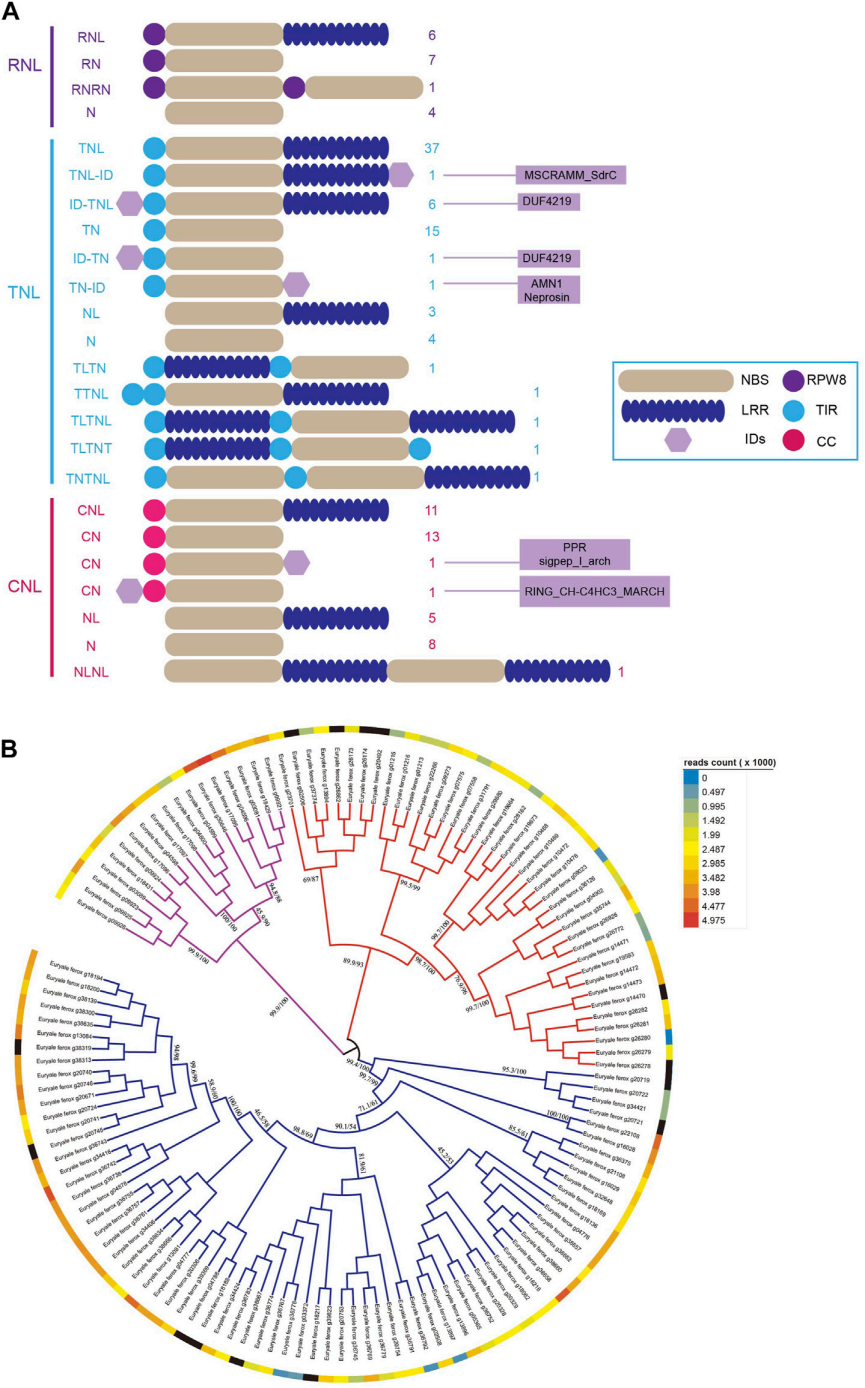
***NBS-LRR*** genes in ***E. ferox*** and their domain combinations. **(A)** Twenty-four domain combinations of *NBS-LRR* genes in *E. ferox*. The numbers of *NBS-LRR* genes of each domain combination are listed and the IDs are also listed as a hexagon. **(B)** The expression profile of 131 *NBS-LRR* genes in *E. ferox*.

Our previous study found that not all *NBS-LRR* genes possess the entire structure, and some *NBS-LRR* genes may lose part of their domains or fuse additional domains during duplication (Shao et al., 2014; Zhang et al., 2016). Based on the domain combinations of the translated proteins, the *NBS-LRR* genes in each subclass were classified into different groups (Figure 1A). The 18 *RNL* genes were divided into four groups according to the structure of proteins. Among them, six intact *RNL* genes accounted for 33.3%. Seven *RNL* genes encoded proteins that lacked the LRR domain, whereas four *RNL* genes encoded proteins that lacked both RPW8 and LRR domains. Additionally, we found an *RNL* gene encoding an *RPW8-NBS-RPW8-NBS* (*RNRN*) structure protein. The structures of *TNL* and *CNL* genes are both more diverse than those of *RNL* genes. We explored 13 different kinds of structures in 73 *TNL* genes and seven kinds of structures in the 40 *CNL* genes. Notably, these structural variations were caused not only by the loss of the typical domains of *NBS-LRR* genes but also by the fusing of additional domains. For example, we found four different integrated domains (IDs) in nine *TNL* genes. Among them, seven IDs were fused to the C-terminal domain of *NBS-LRR* proteins, whereas two were fused to the N-terminal domain. Moreover, transcription analysis indicated that the majority of *NBS-LRR* genes of *E. ferox* were expressed at a low level (Figure 1B; Supplementary Table S2) because they always remain silenced but highly expressed during pathogen stimulation.

### Chromosomal Distribution of *Euryale ferox NBS-LRR* Genes

**FIGURE 2:**
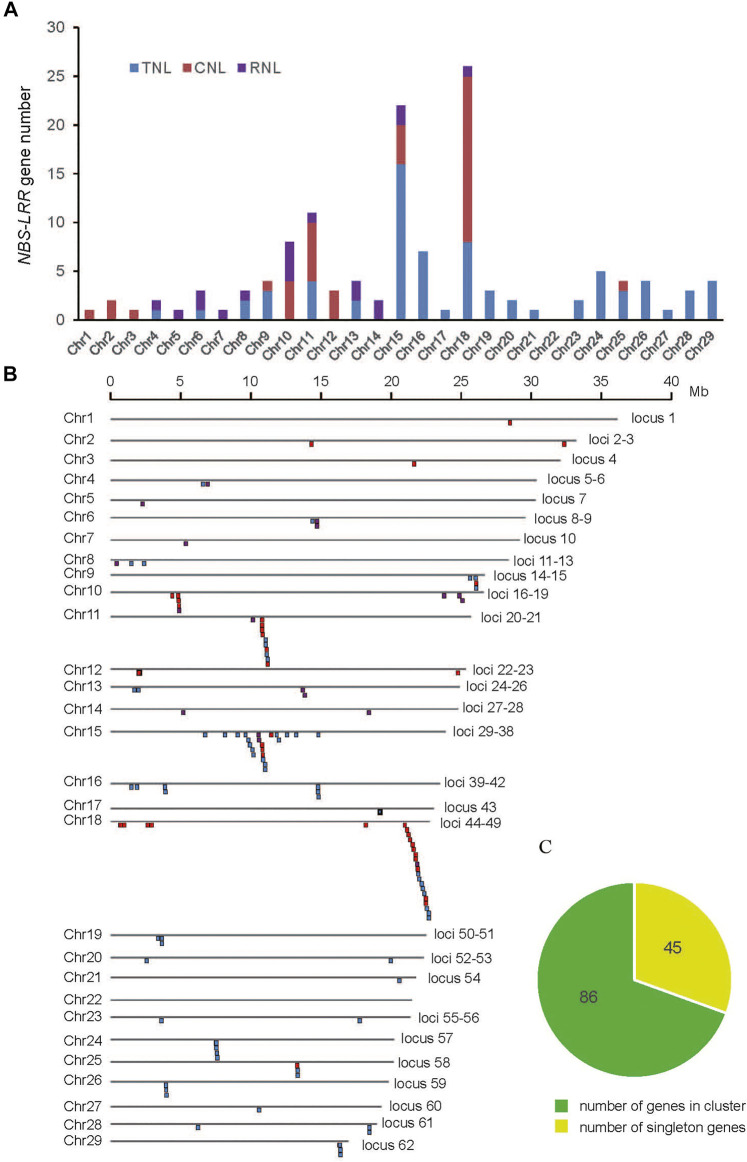
**(A)** The histogram shows the number of *NBS-LRR* genes in each chromosome. **(B)** Chromosomal distribution of *E. ferox NBS-LRR* genes. **(C)** The pie chart shows the proportion between singleton and clustered *NBS-LRR* genes.

The positions of *NBS-LRR* genes were anchored to chromosomes based on their physical location provided by the GFF3 file, and the 131 identified *NBS-LRR* genes were found to be scattered unevenly on 28 of 29 *E. ferox* chromosomes (Figure 2). Chromosomes 11, 15, and 18 had more than 10 *NBS-LRR* genes, whereas only one *NBS-LRR* gene was identified on chromosomes 1, 3, 5, 7, 17, 21, and 27. No *NBS-LRR* genes were detected on chromosome 22. It seems that *NBS-LRR* gene number is not correlated to the length of a chromosome (Supplementary Figure S1).

According to the physical locations, the *NBS-LRR* genes on the 28 chromosomes were classified into 62 loci, including 44 singletons (one gene at one locus) and 18 multigene clusters (Figure 2). The results demonstrated that 87 *NBS-LRR* genes were organized into 18 clusters, which were distributed in chromosomes 11, 15, and 18. On average, there were five genes per cluster. These clusters were mainly distributed on chromosomes 11, 15, and 18, resulting in a high density of *NBS-LRR* genes on these chromosomes. Among the 18 clusters, the smallest one only contained two adjacent genes, while the largest one on chromosome 18 contained 10 genes.

### Different Types of Duplications of *Euryale ferox NBS-LRR* Genes

**FIGURE 3:**
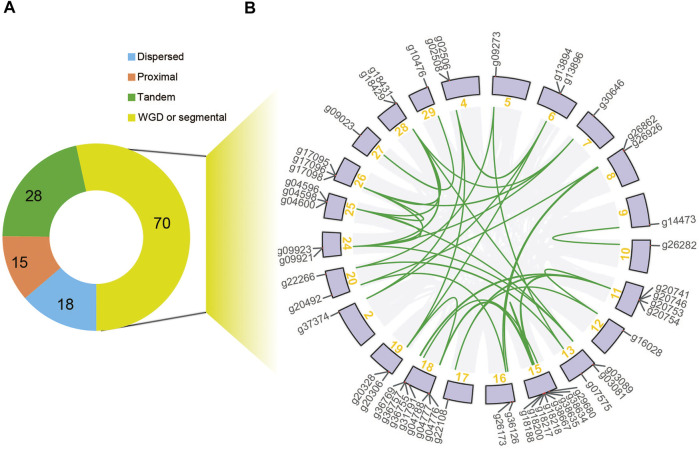
Duplication type of *E. ferox NBS-LRR* genes. **(A)** A pie chart showing *NBS-LRR* genes with different duplication types. **(B)** Syntenic relationships of the 70 segmental-duplicated *NBS-LRR* genes.

The expansion in the *NBS-LRR* gene family was derived from different duplication types, among which a large proportion of *NBS-LRR* genes were organized into clusters due to tandem duplication. Through the analysis of the duplication types of *NBS-LRR* genes, we found that 28 of the 131 genes were produced via tandem duplication, 18 were produced by dispersed duplication, 15 were produced by proximal duplication, and 70 were resulted from segmental duplications or whole-genome duplications (WGD), which were distributed mainly on chromosomes 11, 15, and 18 (Figure 3; Supplementary Figure S2). For instance, gene *g26862* on chromosome eight is syntenic to *g20492* on chromosome 20, so these two genes were considered to be derived from a WGD or a segmental duplication.

### Phylogeny of *NBS-LRR* Genes in *Euryale ferox*

**FIGURE 4:**
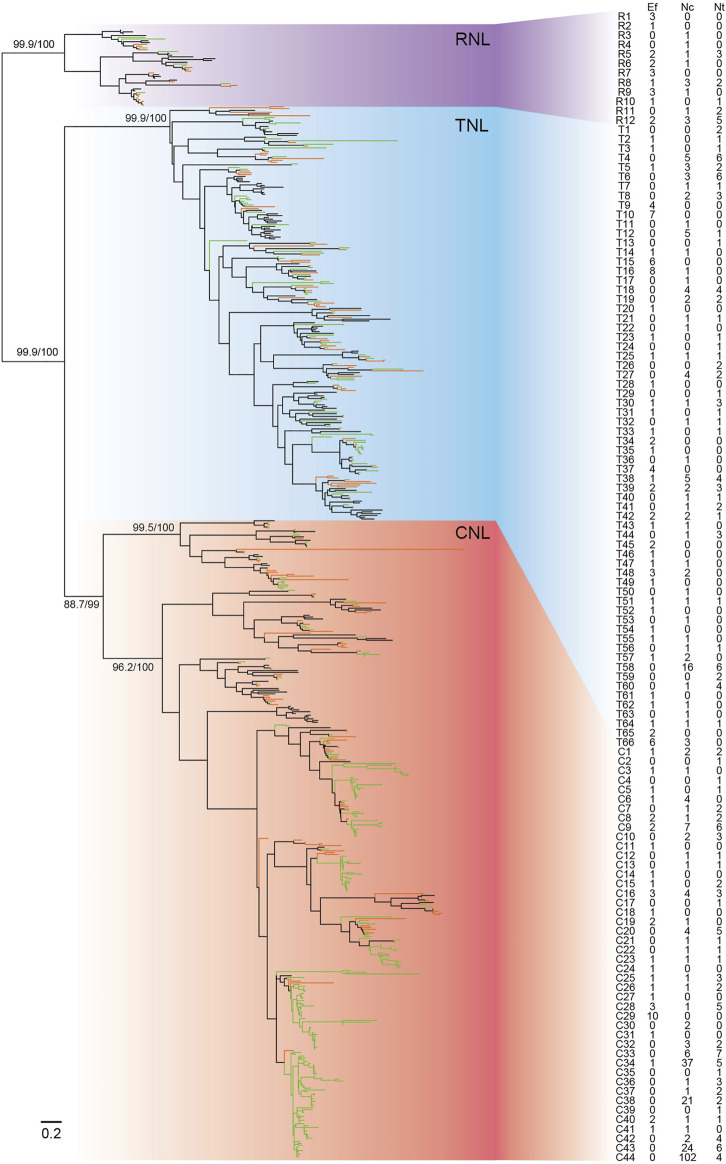
Phylogeny of *NBS-LRR* genes of *E. ferox, N. colorata*, and *N. thermarum*. The phylogeny was constructed based on the conserved NBS domain of *NBS-LRR* genes from *E. ferox* (Ef, green branches), *N. colorata* (Nc, orange branches), and *N. thermarum* (Nt, black branches). Branch support values obtained from a UFBoot2 test are labeled on basal nodes. Predicted ancestral lineages are labeled with numbers. The detailed phylogenetic tree is shown in Supplementary Figure S1.

To trace the evolutionary history of *NBS-LRR* genes in *E. ferox*, a phylogenetic tree was constructed together with *NBS-LRR* genes from two species in *Nymphaeaceae*, *N. colorata* and *N. thermarum*, and *Arabidopsis thaliana*. The phylogenetic tree consisted of three monophyletic clades, *CNL*, *TNL*, and *RNL*, with high support values (Figure 4). The *RNL* monophyletic clade in the phylogenetic tree had a high support value (99.9%). The topology was consistent with that of other investigated angiosperms, supporting the notion that the three subclasses diverged in early ages (Xue et al., 2012; Shao et al., 2019).

Based on the reconstructed *NBS-LRR* gene phylogeny, 122 ancestral *NBS-LRR* lineages in the common ancestor of the three species (including 44 *CNL*s, 66 *TNL*s, and 12 *RNL*s) were recovered (Figure 4). Clusters organized by several *NBS-LRR* genes from *E. ferox* and two water lilies were detected in the phylogenetic tree, which mirrored the species-specific expansion, especially the drastic expansion of *CNL*s in *N. colorata* in the C44 lineage, with 102 *CNL*s. Of 122 ancestral lineages, *E. ferox* inherited 70 lineages, whereas there were only 131 *NBS-LRR* genes in the *E. ferox* genome, suggesting that a fairly low level of *NBS-LRR* gene expansion occurred in the *E. ferox* genome during evolution.

## Discussion

*NBS-LRR* genes consist of hundreds of members and are the largest family among *R* genes. In two-tier immune systems, the *NBS-LRR* gene family plays important roles in effector-triggered immunity. Revealing the profile of *NBS-LRR* genes in a species is very important, and by exploring the evolutionary patterns of *NBS-LRR* genes in the species and its close relatives, we can put these conclusions into practice (Meyers et al., 2003; Zhang et al., 2016). In the past 20 years, genome-wide identification and evolutionary analysis have been conducted in many angiosperms (Liu et al., 2021; Luo et al., 2012; Qian et al., 2017; Shao et al., 2014; Xue et al., 2020; Zhang et al., 2016; Zhou et al., 2020). It is noteworthy that of the more than 300 disease resistance genes, over 60% that have been cloned from different plants belong to the *NBS-LRR* gene family (Kourelis and van der Hoorn, 2018). Angiosperms inherited three subclasses of *NBS-LRR* genes: *TNL*s, *CNL*s, and *RNL*s. Studies have shown that proteins encoded by *CNL*s and *TNL*s function as receptors for pathogen recognition, and these two subclasses have expanded to various degrees during the long-term ‘arms race’ with pathogens (Shao et al., 2014).

In this study, the *E. ferox* genome was found to harbor only 131 *NBS-LRR* genes, consistent with a recent report. Liu et al. (2021) proposed that a convergent contraction of *NBS-LRR* genes occurred during the evolution of adaptation to an aquatic lifestyle, which may be the reason for the fewer *NBS-LRR* genes in *E. ferox* compared to other angiosperms. The structures of *NBS-LRR* genes are relatively diverse. Among four different IDs, MARCH1, also known as membrane-associated RING finger protein 1, is a membrane-anchored E3 ubiquitin ligase that mainly expressed in cells of the immune system (Bartee et al., 2004). MARCH1 also plays a regulatory role in T cell activation during immune responses, thus, we speculated that it regulates immune responses in plants.

*NBS-LRR* genes can expand via different duplication methods. Many studies have shown that tandem duplication is dominant in *NBS-LRR* gene expansion in most angiosperms (Shao et al., 2014; Qian et al., 2017; Zhou et al., 2020). We analyzed the duplication types of *NBS-LRR* genes in the *E. ferox* genome and found that there were 28 *NBS-LRR* genes generated by tandem duplication in the *E. ferox* genome, accounting for only 21% in total. This proportion is much lower than the 70–80% in the legume family (Shao et al., 2014). Furthermore, it was documented that *E. ferox* experienced two rounds of WGDs (Yang et al., 2020). Thus, we thought since tandem duplications were rare in its evolutionary history, the majority of *E. ferox NBS-LRR* genes are derived from segmental duplications, which is different from the situation in most other angiosperms, and the fewer number of tandemly duplicated genes may also be one of the reasons for the relatively fewer *NBS-LRR* genes in *E. ferox*. Additionally, the phylogenetic analysis of *NBS-LRR* genes in *E. ferox* and two other Nymphaeaceae species revealed that there were at least 122 ancestral *NBS-LRR* genes in the common ancestor of the three species, whereas only 131 *NBS-LRR* genes were found in the *E. ferox* genome, which further indicated a low level of *NBS-LRR* gene expansion in the *E. ferox* genome during its long-term evolution.

The comprehensive identification of *NBS-LRR* genes from genomic data is not only important for revealing the evolution of this important disease resistance gene family but also serves as an important basis for discovering and utilizing functional disease resistance genes in crops. Based on the analysis of *NBS-LRR* genes in the rice genome, Zhang et al. (2015) used targeted cloning of *NBS-LRR* genes in resistant rice varieties, and verified dozens of *NBS-LRR* genes that play important roles in blast resistance in rice. Witek et al. (2016) successfully cloned the late blight resistance gene Rpi-AMR3I from the resistance locus of *Solanum americanum* by target region sequencing and third-generation sequencing based on the analysis of *NBS-LRR* genes in the potato genome. These studies indicate that the genome-wide identification and analysis of *NBS-LRR* genes promotes the cloning of functional resistance genes in the studied species and related species. *E. ferox* is an important functional food and medicinal plant that has been widely cultivated in China since time immemorial. However, due to its characteristics of underwater flowering and pollination, research on germplasm innovation and disease resistance breeding has lagged behind research in other species. With the breakthrough of artificial hybrid technology for *E. ferox* in the 1980s and the development of tissue culture technology in recent years, it is possible to breed superior varieties of *E. ferox* by molecular breeding. *E. ferox* is vulnerable to a large number of diseases, so it is particularly important to develop molecular breeding in *E. ferox*.

In summary, the present study identified a complete set of 131 *NBS-LRR* genes in the genome of *E. ferox*. Although the quantity is significantly lower than that of most species in Solanaceae, Leguminosae, and Poaceae, it is roughly equivalent to that of Brassicaceae crops, and higher than those of Cucurbitaceae crops (Luo et al., 2012; Lin et al., 2013; Shao et al., 2014; Morata and Puigdomènech, 2017; Qian et al., 2017). The identification of *NBS-LRR* genes, analysis of sequence features, and revelation of chromosomal distribution pattern will provide important references for the molecular breeding of *E. ferox*. In addition, Nymphaeaceae contains many kinds of important flowering plants. The comparative analysis of *NBS-LRR* genes in these plants will also facilitate the molecular breeding of different species in the same genus.

## Data Availability

The original contributions presented in the study are included in the article/Supplementary Material, further inquiries can be directed to the corresponding authors.
