# Supplementary material for: Genome-Wide Analysis of NBS-LRR Genes From an Early-Diverging Angiosperm Euryale ferox
Source: Front Genet. 2022 May 13;13:880071. doi: 10.3389/fgene.2022.880071 (PMC9140740; doi:10.3389/fgene.2022.880071)
Supplement: Supplementary file 1 [file DataSheet2.pdf]

Supporting information

Figure S1 Scatter plot of correlation analysis between *NBS-LRR* gene number and the length of a chromosome.

Figure S2 Dotplot showing syntenic blocks (involving ≥10 colinear genes) in *E. ferox* genome.

Supporting information:

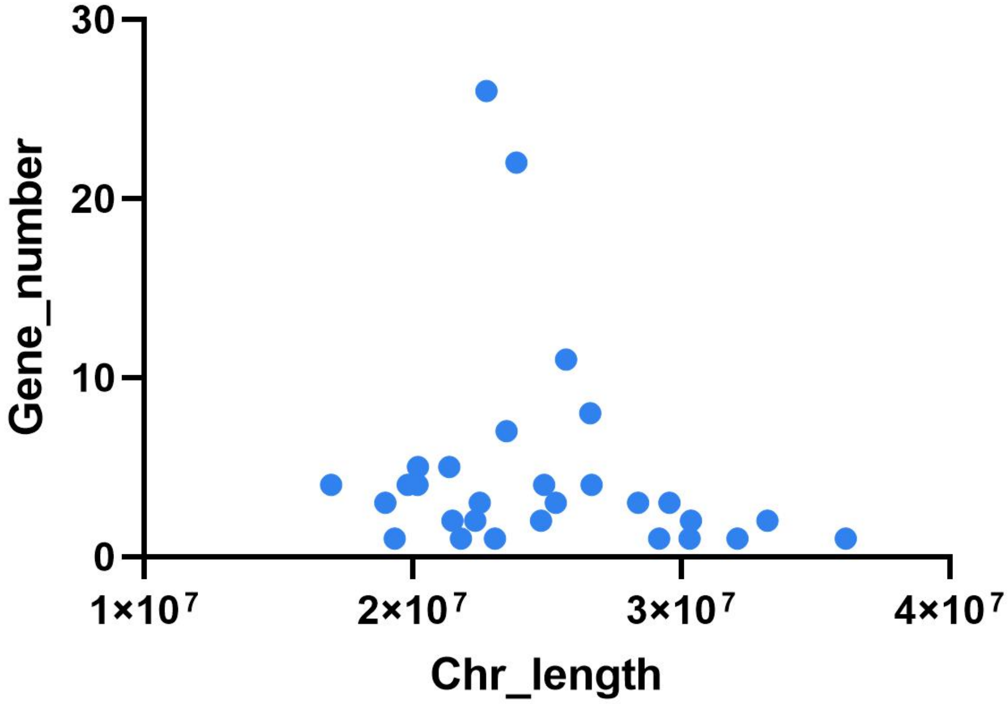

Figure S1 Scatter plot of correlation analysis between *NBS-LRR* gene number and the length of a chromosome.

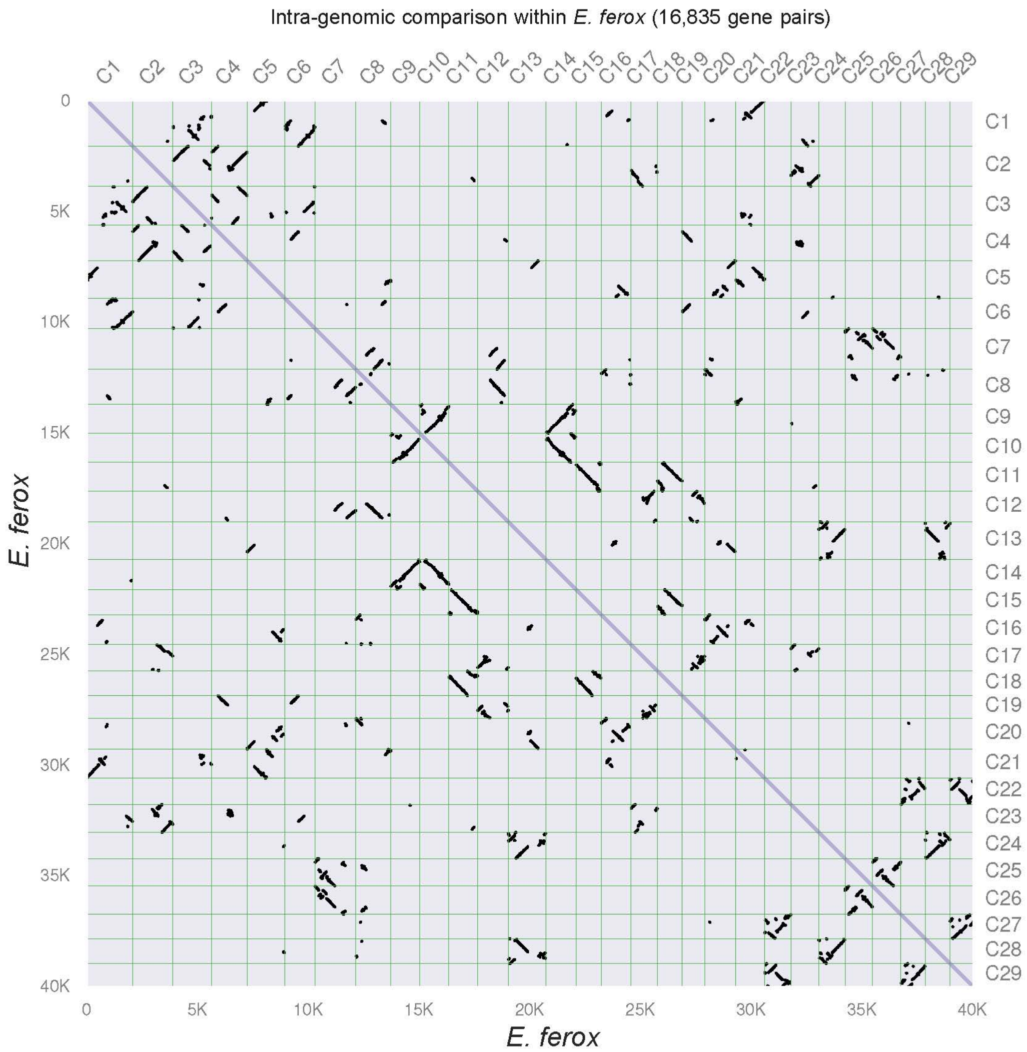

Figure 2 Dotplot showing syntenic blocks (involving ≥10 colinear genes) in *E. ferox* genome.
