# Supplementary material for: Genome-Wide Analysis of NBS-LRR Genes From an Early-Diverging Angiosperm Euryale ferox
Source: Front Genet. 2022 May 13;13:880071. doi: 10.3389/fgene.2022.880071 (PMC9140740; doi:10.3389/fgene.2022.880071)
Supplement: Supplementary file 2 [file DataSheet1.docx]

**Supporting information**

**Table S1** A list of *NBS-LRR* genes identified from *E. ferox.*

**Table S2** Expression of *NBS-LRR* genes in the *E.ferox*.

**Table S1** A list of *NBS-LRR* genes identified from *E. ferox.*

| Gene ID | Classification | Domain composition | Duplication Type |
| --- | --- | --- | --- |
| g01213 | CNL | CN | Proximal |
| g01215 | CNL | CNL | Tandem |
| g01216 | CNL | CN | Tandem |
| g02506 | CNL | NL | WGD or Segmental |
| g02508 | TNL | TNL | WGD or Segmental |
| g03081 | RNL | RNL | WGD or Segmental |
| g03089 | RNL | RN | WGD or Segmental |
| g03372 | TNL | TNL | Dispersed |
| g04596 | RNL | RN | WGD or Segmental |
| g04598 | RNL | RNL | WGD or Segmental |
| g04599 | RNL | N | Tandem |
| g04600 | RNL | N | WGD or Segmental |
| g04776 | TNL | TTNL | WGD or Segmental |
| g04777 | TNL | TNL | WGD or Segmental |
| g04788 | TNL | N | WGD or Segmental |
| g04878 | TNL | TNL | Dispersed |
| g04902 | CNL | CNL | Dispersed |
| g05365 | TNL | TNL | Dispersed |
| g07558 | CNL | CN | WGD or Segmental |
| g07575 | CNL | CNL | WGD or Segmental |
| g09023 | CNL | CNL | WGD or Segmental |
| g09273 | CNL | CN | WGD or Segmental |
| g09921 | RNL | RN | WGD or Segmental |
| g09923 | RNL | RN | WGD or Segmental |
| g09924 | RNL | RN | Tandem |
| g09925 | RNL | N | Tandem |
| g09926 | RNL | N | Tandem |
| g10468 | CNL | RING-CN | Dispersed |
| g10469 | CNL | NL | Proximal |
| g10472 | CNL | CNL | Proximal |
| g10476 | CNL | CNL | WGD or Segmental |
| g13081 | TNL | TNL | Dispersed |
| g13084 | TNL | DUF-TNL | Dispersed |
| g13894 | CNL | NL | WGD or Segmental |
| g13896 | TNL | TN | WGD or Segmental |
| g13897 | TNL | N | Tandem |
| g14470 | CNL | N | Proximal |
| g14471 | CNL | CN | Tandem |
| g14472 | CNL | CN | Tandem |
| g14473 | CNL | N | WGD or Segmental |
| g16028 | TNL | TN | WGD or Segmental |
| g16029 | TNL | TNL | WGD or Segmental |
| g17095 | RNL | RNL | WGD or Segmental |
| g17096 | RNL | RN | WGD or Segmental |
| g17097 | RNL | RN | Tandem |
| g17098 | RNL | RNL | WGD or Segmental |
| g18062 | TNL | TNL | Dispersed |
| g18136 | TNL | TNL | Dispersed |
| g18169 | TNL | TNL | Dispersed |
| g18188 | TNL | TNTNL | WGD or Segmental |
| g18194 | TNL | TN | WGD or Segmental |
| g18200 | TNL | TN | WGD or Segmental |
| g18217 | TNL | TN | WGD or Segmental |
| g18218 | TNL | TN | WGD or Segmental |
| g18429 | RNL | RNL | WGD or Segmental |
| g18431 | RNL | RNRN | WGD or Segmental |
| g19593 | CNL | CN | WGD or Segmental |
| g19664 | CNL | CNL | Proximal |
| g19673 | CNL | CNL | Dispersed |
| g20306 | TNL | TNL | WGD or Segmental |
| g20328 | TNL | NL | WGD or Segmental |
| g20329 | TNL | TNL | Tandem |
| g20492 | CNL | N | WGD or Segmental |
| g20671 | TNL | DUF-TNL | Dispersed |
| g20719 | TNL | TN | Tandem |
| g20721 | TNL | LN | WGD or Segmental |
| g20722 | TNL | TN | Proximal |
| g20724 | TNL | TN | Proximal |
| g20740 | TNL | TNL | Tandem |
| g20741 | TNL | TNL | WGD or Segmental |
| g20745 | TNL | TN | Tandem |
| g20746 | TNL | TNL | WGD or Segmental |
| g20753 | TNL | TN | WGD or Segmental |
| g20754 | TNL | TNL | WGD or Segmental |
| g21108 | TNL | TNL | WGD or Segmental |
| g22108 | TNL | TN | WGD or Segmental |
| g22266 | CNL | CN | WGD or Segmental |
| g23701 | CNL | NL | WGD or Segmental |
| g26173 | CNL | N | WGD or Segmental |
| g26174 | CNL | NNL | Tandem |
| g26278 | CNL | N | Tandem |
| g26279 | CNL | CN | Tandem |
| g26280 | CNL | N | Tandem |
| g26281 | CNL | CN | Tandem |
| g26282 | CNL | CN | WGD or Segmental |
| g26772 | CNL | CN | Tandem |
| g26862 | CNL | N | WGD or Segmental |
| g26926 | CNL | CNL | WGD or Segmental |
| g28162 | CNL | CN | Dispersed |
| g29680 | CNL | CNL | WGD or Segmental |
| g30646 | RNL | RNL | WGD or Segmental |
| g31791 | CNL | N | WGD or Segmental |
| g32648 | TNL | TNL | Dispersed |
| g34406 | TNL | DUF-TNL | Tandem |
| g34416 | TNL | TNL | Proximal |
| g34421 | TNL | TNL | WGD or Segmental |
| g34424 | TNL | TNL | Tandem |
| g35744 | CNL | CN | Dispersed |
| g36126 | CNL | CNL | WGD or Segmental |
| g36375 | TNL | TNL | Tandem |
| g36738 | TNL | TNL | WGD or Segmental |
| g36742 | TNL | TNL | Proximal |
| g36743 | TNL | TNL | Proximal |
| g36745 | TNL | NL | Tandem |
| g36752 | TNL | TNL | Dispersed |
| g36755 | TNL | DUF-TNL | WGD or Segmental |
| g36757 | TNL | TNL | WGD or Segmental |
| g36761 | TNL | DUF-TNL | WGD or Segmental |
| g36767 | TNL | TLTN | WGD or Segmental |
| g36769 | TNL | TN | WGD or Segmental |
| g36774 | TNL | TLTNL | Proximal |
| g36778 | TNL | TLTNT | Proximal |
| g36779 | TNL | TNL | WGD or Segmental |
| g36783 | TNL | TN | Proximal |
| g36791 | TNL | TNL | Tandem |
| g36792 | TNL | TNL | Tandem |
| g37374 | CNL | NL | WGD or Segmental |
| g38139 | TNL | TNL | Tandem |
| g38300 | TNL | DUF-TNL | WGD or Segmental |
| g38313 | TNL | TNL | WGD or Segmental |
| g38319 | TNL | TN | WGD or Segmental |
| g38634 | TNL | TNL | WGD or Segmental |
| g38635 | TNL | TNL | WGD or Segmental |
| g38657 | TNL | TNL | WGD or Segmental |
| g38658 | TNL | TNL | WGD or Segmental |
| g38660 | TNL | TN-ID | Tandem |
| g38662 | TNL | N | Proximal |
| g38666 | TNL | DUF-TN | Proximal |
| g38667 | TNL | N | WGD or Segmental |
| g39369 | TNL | TNL | Dispersed |
| g39823 | TNL | TNL-ID | Dispersed |
| g01213 | CNL | CN | Proximal |

**Table S2** Expression of *NBS-LRR* genes in the *E.ferox*.

| Gene ID | Read Counts |
| --- | --- |
| g19664 | 192 |
| g09273 | 729 |
| g22266 | 41 |
| g26173 | 119 |
| g31791 | 714 |
| g10468 | 1316 |
| g20492 | 0 |
| g01213 | 36 |
| g37374 | 19 |
| g14473 | 3174 |
| g09023 | 2 |
| g10469 | 746 |
| g26278 | 0 |
| g36126 | 122 |
| g01216 | 149 |
| g02506 | 0 |
| g26174 | 0 |
| g14472 | 2137 |
| g28162 | 11 |
| g07558 | 19 |
| g26279 | 168 |
| g23701 | 672 |
| g14471 | 6 |
| g04902 | 73 |
| g26772 | 8 |
| g26862 | 0 |
| g13894 | 309 |
| g10476 | 811 |
| g26926 | 423 |
| g07575 | 290 |
| g26281 | 1964 |
| g26280 | 1 |
| g26282 | 580 |
| g01215 | 12 |
| g14470 | 0 |
| g10472 | 223 |
| g19593 | 1586 |
| g35744 | 2904 |
| g19673 | 283 |
| g29680 | 515 |
| g09925 | 162 |
| g18431 | 1790 |
| g18429 | 2191 |
| g17096 | 8598 |
| g04599 | 380 |
| g04598 | 5363 |
| g09921 | 5472 |
| g03081 | 3173 |
| g17098 | 1679 |
| g03089 | 270 |
| g30646 | 49238 |
| g17097 | 1485 |
| g09924 | 70 |
| g04600 | 27 |
| g17095 | 94394 |
| g04596 | 22881 |
| g09923 | 6586 |
| g09926 | 304 |
| g03372 | 3 |
| g20721 | 0 |
| g38313 | 6600 |
| g20328 | 416 |
| g34424 | 2501 |
| g38139 | 1339 |
| g34416 | 3153 |
| g13084 | 16757 |
| g38635 | 3473 |
| g38667 | 0 |
| g05365 | 107 |
| g13897 | 2 |
| g16029 | 1531 |
| g20754 | 483 |
| g18194 | 6394 |
| g20329 | 135 |
| g20753 | 244 |
| g18218 | 347 |
| g38658 | 44333 |
| g36774 | 91 |
| g34421 | 10 |
| g36783 | 350 |
| g04788 | 0 |
| g38660 | 4739 |
| g38666 | 254 |
| g18062 | 320 |
| g36752 | 236 |
| g36792 | 2 |
| g04878 | 6713 |
| g36757 | 9250 |
| g32648 | 781 |
| g36767 | 556 |
| g36755 | 6600 |
| g20722 | 10 |
| g13896 | 2324 |
| g36745 | 26 |
| g39369 | 1036 |
| g18200 | 4286 |
| g04776 | 4371 |
| g20724 | 277 |
| g36778 | 2 |
| g22108 | 31951 |
| g38662 | 436 |
| g36375 | 3315 |
| g36742 | 59339 |
| g18169 | 713 |
| g18188 | 0 |
| g36743 | 8339 |
| g18136 | 4463 |
| g39823 | 1526 |
| g20745 | 0 |
| g20746 | 16797 |
| g36779 | 84 |
| g38319 | 0 |
| g20719 | 0 |
| g18217 | 0 |
| g38634 | 1434 |
| g20306 | 4623 |
| g20671 | 5679 |
| g20741 | 1261 |
| g36769 | 4046 |
| g20740 | 5973 |
| g38657 | 4606 |
| g36761 | 7349 |
| g04777 | 5975 |
| g13081 | 27604 |
| g16028 | 10803 |
| g36738 | 8578 |
| g02508 | 1065 |
| g21108 | 630 |
| g34406 | 6388 |
| g36791 | 460 |
| g38300 | 5283 |
